# Preparation and Chemical Protective Clothing Application of PVDF Based Sodium Sulfonate Membrane

**DOI:** 10.3390/membranes10080190

**Published:** 2020-08-17

**Authors:** Yue Zhao, Xinbo Wang, Deyin Wang, Heguo Li, Lei Li, Shouxin Zhang, Chuan Zhou, Xiaohui Zheng, Quanfu Men, Jinyi Zhong, Liang Wu

**Affiliations:** 1 State Key Laboratory of NBC Protection for Civilian, Institute of Chemical Defense, Beijing 100191, China; SA11226532@mail.ustc.edu.cn (Y.Z.); wxb1993@mail.ustc.edu.cn (X.W.); xpz-wdy@163.com (D.W.); liheguo1972@126.com (H.L.); lanepku@126.com (L.L.); zhang_shouxin_love@126.com (S.Z.); zhouc_fh@163.com (C.Z.); zheng_nudt@163.com (X.Z.); 2 College of Chemistry and Materials, University of Science and Technology of China, Hefei 230026, China

**Keywords:** chemical protection, sodium sulfonate membrane, nerve agents, water vapor permeation, membrane separation

## Abstract

Chemical protective clothing (CPC) is major equipment to protect human skin from hazardous chemical warfare agents (CWAs), especially nerve agents and blister agents. CPC performance is mainly dominated by the chemical protective material, which needs to meet various requirements, such as mechanical robustness, protective properties, physiological comfort, cost-effectiveness, and dimensional stability. In this study, polyvinylidene fluoride (PVDF) based sodium sulfonate membranes with different ion exchange capacities (IECs) are prepared simply from low-cost materials. Their mechanical properties, contact angles, permeations, and selectivities have been tested and compared with each other. Results show that membranes with IEC in the range of 1.5–2 mmol g^−1^ have high selectivities of water vapor permeation over CWA simulant vapor permeation and good mechanical properties. Therefore, PVDF-based sodium sulfonate membranes are potential materials for CPC applications.

## 1. Introduction

**Figure 1:**
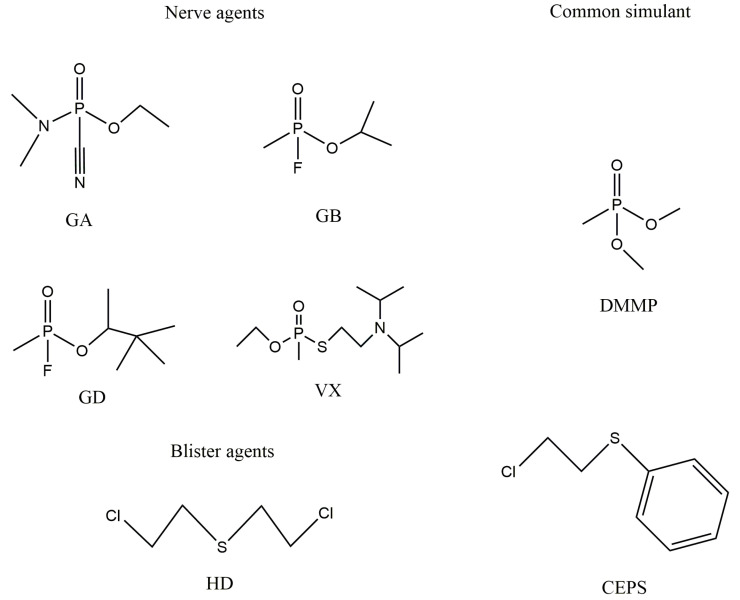
Common CWAs and their simulants.

Nerve agents are the most toxic class of chemical warfare agents (CWAs), including tabun (GA), sarin (GB), soman (GD), and VX as representatives (Figure 1). These agents are usually colorless, odorless, and highly toxic. Vapor permeation is the primary route of exposure to nerve agents, which can cause immediate damage after exposure to small amounts of nerve agents vapor [1]. Sulfur mustard, one of the most famous CWAs introduced in World War I, can easily permeate the human skin and alkylate the DNA to cause cell death. Sulfur mustard as the representative of blister agents has been widely used in many wars during the last century and caused thousands of deaths [2]. Therefore, it is crucial to develop chemical protective clothing (CPC) to protect people from exposure to CWAs vapors. In the initial stage, researchers use simulants to study and evaluate the protective performance against nerve agents. The most commonly used simulants are dimethyl methyl phophonate (DMMP) for nerve agents and 2-chloroethyl phenyl sulfide (CEPS) for blister agents (Figure 1).

Rubbery barrier materials have been previously demonstrated as effective CPC materials against CWAs [3,4,5]. However, the transfer of water vapor was blocked at the same time, resulting in heat stress and faint for the users [6]. On the other hand, adsorptive fabrics with activated carbon adsorbent have also been used to adsorb chemical warfare agents [7,8]. Although better physiological comfort could be achieved, there still exist defects, such as limited sorbent capacity, high weight, and secondary contamination caused by desorption [9]. Ideally, the protective material should work selectively, which transports water and blocks water-soluble toxic compounds. Polyelectrolyte membranes (PEMs) composed of hydrophobic and hydrophilic segments may be used to resolve this apparent contradiction. The self-assembly of a PEM upon solvation can form a permselective network containing nanoscale hydrophilic channels. Typical examples of such selective permeable membranes are sulfonated poly(styrene-isobutylene-styrene) (SIBS), triblock copolymer, perfluorinated ionomers, and polystyrene sulfonic acid (PSSA) [10,11,12,13,14,15,16,17,18,19]. However, sulfonated SIBS has poor selectivity between 0.7 and 35 [19], Nafion is too expensive for chemical protective clothing usage ($1600 m^−2^ for Nafion 117) [20], and polystyrene sulfonic acid has a poor mechanical performance [14]. As for the selectivity of the membrane, Suleiman et al. systematically studied the water/DMMP selectivity of a SIBS-based ion exchange membrane under different degrees of sulfonation [18,19]. By using classical molecular dynamics simulations, Neimark et al. explored the nanoscale segregation and diffusion of water and nerve gas simulant DMMP in sulfonated polystyrene (sPS) at different sulfonation and hydration levels. The result suggests that sPS is permeable for both DMMP and water. However, DMMP molecules partially segregated on the scale of 1–2 nm and have different pathways through the system [15]. Therefore, a new membrane design concept for CPC application, and deepening understanding of the relationship between membrane structure and selective transport properties, is desirable.

Building on recent advances with ion-exchange membranes, modification based on existing commercial high-performance polymers can reduce costs and obtain excellent and stable membrane performances. Polyvinylidene chloride (PVDC) is well known as an ideal packaging material with high barrier properties, strong toughness, excellent chemical stability, and low thermal shrinkage. Notably, it has a high tolerance to acid, alkali, oil immersion, and various chemical solvents [21,22]. However, its low-temperature solubility, poor compatibility with hydrophilic additives limit its wide application for CPC. Compared with PVDC, polyvinylidene fluoride (PVDF) has more advantages in high chemical stability and excellent barrier and solution-casting properties. Therefore, PVDF has broad application prospects as a matrix resin used in a CPC selectively permeable membrane and is chosen for this study [23,24,25,26,27,28].

PVDF can usually be modified by using high-energy radiation, inorganic strong base to obtain modified PVDF films containing active sites or carbon–carbon double bonds [27,29,30,31]. However, radiation sources are required in the high-energy radiation method. Moreover, inorganic alkali is difficult to permeate into the PVDF membrane. Both methods can only modify the surface of the PVDF membrane and lead to a low water vapor transmission rates (WVTR). Additionally, PVDF can also be grafted with functional polymers in organic solvents by using ozone-induced graft copolymerization and atom transfer radical polymerization (ATRP). However, for ozone-induced graft copolymerization, an ozone generator is required and leads to safety hazards [32,33]. For the ATRP method, transition metal complex inorganic substances are required in the preparation process and difficult to be removed, lead to low blockage of chemical agent simulants. Most importantly, the ATRP method requires a strict anhydrous and oxygen-free environment, which is difficult to satisfy in a large-scale application [34]. Tetramethylammonium hydroxide (TMAH) is a strong organic alkali, which can attack alkane halide to generate carbon–carbon double bonds by the elimination of hydrogen halide through elimination reaction. Moreover, the TMAH methanol solution and its decomposition products (methanol and trimethylamine) have a low boiling point, which can be completely removed at a specific temperature without affecting the modification effect. Thus, the PVDF modification of using TMAH is an ideal method to take into account the cost, simplicity, and post-treatment for CPC membrane preparation.

In this study, a functional polymer, poly(vinylidene fluoride)-graft-poly(sodium p-styrene sulfonate)(PVDF-*g*-SSS) was synthesized via one-pot reaction involving PVDF modification and sodium p-styrene sulfonate (SSS) grafting. The synthetic procedure for the PVDF-*g*-SSS was synthesized under convenience and started from low costing materials. The ion exchange capacity (IEC, molar of the sulfonic acid groups per gram of dried membranes) could be finely tuned in the range of 0 to 2.29 mmol/g. After membrane preparation, the obtained PVDF-*g*-SSS was evaluated as the membrane material for CPC application. The effect of IEC on mechanical properties, contact angles, permeations, and selectivities were systematically investigated. It was found that the IEC is the dominating factor that decides the selectivity and mechanical properties [35]. At an optimized IEC value of 1.5–2 mmol/g, the PVDF-*g*-SSS showed good CPC performance.

## 2. Materials and Methods

### 2.1. Materials

Dimethyl methyl phophonate (DMMP), 2-Chloroethyl phenyl sulfide (CEPS), 2,2-azobis(2-methylpropionitrile) (AIBN), divinylbenzene (DVB, cross-linking agent), tetramethylammonium hydroxide (TMAH), methanol, sodium p-styrene sulfonate (SSS), poly(vinylidene fluoride) (PVDF), and *N*,*N*-dimethylformamide (DMF) were purchased from Sigma–Aldrich (Merck KGaA, Darmstadt, Germany).

### 2.2. Membranes Preparation

PVDF (20 g) was dissolved in DMF, and the amount of DMF is shown in Table 1. The mixture was then stirred at 50 °C to obtain a uniform, transparent solution. Under the nitrogen atmosphere, the PVDF solution was stirred at 50 °C for 1.5 h after adding 1.0 mL of tetramethylammonium hydroxide methanol solution. Afterward, a different amount of SSS (0 g, 2.5 g, 6 g, 10.4 g, 16 g, 24 g) was dissolved in the above solution at 50 °C to get tunable IEC values. DVB crosslinker (10 wt% relative to SSS) and AIBN initiator were then added. The mixed solution reacted at 80 °C under the nitrogen atmosphere for 8h. After cooling down to room temperature, the as-prepared polyelectrolyte solution was evenly cast on the glass plates by using a glass rod, and the thickness of the membrane was adjusted by controlling the height of the glass rod from the glass plate (200 um). Finally, the glass plates were dried on a heating plate at 60 °C for 12 h to form the membranes. The obtained dry copolymer membranes were named M1–M6 (corresponding compositions are listed in Table 1).

### 2.3. Infrared Spectrum Analysis

A Nicolet iS10 FT-IR Spectrometer (ThermoFisher Scientific, Waltham, MA, USA) was used to perform the Fourier transform infrared (FT-IR) analysis.

### 2.4. Morphology Analysis

The JEOL JSM-7900F electron microscope (JEOL Ltd., Akishima, Tokyo, Japan) was used to observe the surface and cross-section morphology of the prepared membranes with a test voltage of 20kV.

### 2.5. Water Uptake (WU), Ion-Exchange Capacity (IEC), and Linear Swelling Ratio (LSR)

IEC value was measured by acid-base titration. First, the membranes (0.2 g) were changed to acid form by being immersed in 1 M HCl aqueous solution for 24 h. Secondly, the membranes were washed with DI and weighed by analytical balance. Finally, the membranes were soaked in NaCl aqueous solution (1 M) and equilibrated for 24 h. Hydrogen ions were quantified by titration of 0.1 M NaOH aqueous solution using phenolphthalein as an indicator. The IEC value can be calculated by the mass of the dry membrane in H^+^ form (*W*_dry_) and the amount of NaOH consumed in the titration (*V*_NaOH_ × *C*_NaOH_) (Equation (1)).

(1) $$\mathrm{IEC}=\frac{V_{\left(\mathrm{NaOH}\right)}\times C_{\left(\mathrm{NaOH}\right)}}{W_{\mathrm{dry}}}$$

WU and LSR of the membranes (10 × 30 mm) were calculated by following equations (Equations (2) and (3)) based on the measured data of weight and length of membranes after being immersed in water and equilibrated for 24 h, respectively.

(2) $$\mathrm{WU}=\frac{W_{\mathrm{wet}}-W_{\mathrm{dry}}}{W_{\mathrm{dry}}}\times 100\%$$

(3) $$\mathrm{LSR}=\frac{L_{\mathrm{wet}}-L_{\mathrm{dry}}}{L_{\mathrm{dry}}}\times 100\%$$

where the weight and length of the wet membrane are marked as *W*_wet_ and *L*_wet_, whereas the weight and length of the dry membrane are marked as *W*_dry_ and *L*_dry_.

### 2.6. Water Contact Angle (CA)

To evaluate the surface properties of the obtained membranes, a contact angle meter (SL200B, Solon Tech Co., Ltd., Shenzhen, Guangdong, China) was used to perform static contact angle measurement with DI water at room temperature. Three parallel specimens were measured to ensure the accuracy of the results.

### 2.7. Evaluation of Mechanical Properties of Membranes

An Instron Universal Testing Machine (Instron Co., Canton, MA, USA) was used to measure the mechanical properties of membranes with a certain size (5 mm × 30 mm) by tensile testing at an extension rate of 1 mm s^−1^. Three parallel tests were conducted for each membrane to obtain stress–strain curves, and elongation-at-break and tensile strength were obtained from the stress–strain curves.

### 2.8. Evaluation of Permeations and Selectivities

**Figure 2:**
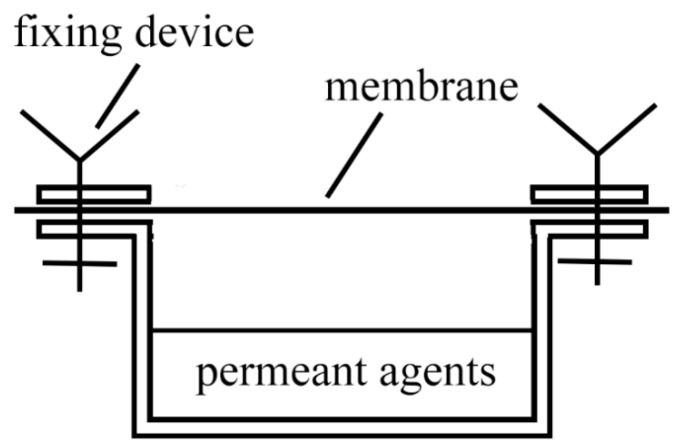
Schematic drawing of the permeation cell.

The American Society for Testing and Materials (ASTM) E96-95 procedure was used to measure the vapor permeation of obtained membranes with a slight modification. Samples to be tested were located over the open-top testing vials with certain mass permeants (water, DMMP, or CEPS) and fixed with fixtures to constitute permeation cells after dried at 60 °C for 24 h, and the thickness was measured with a digital caliper (Figure 2). The effective test area is 6.28 cm^2^. The obtained permeation cells were placed in a convection oven at 35 °C and 10% RH and weighed at certain times. After the weight losses (*W*) became constant, they were calculated from the slopes of the weight–time curves obtained from measuring the weights of permeation cells. Vapor transfer rates (g m^−2^ day^−1^) (*VTR*) and vapor permeation (*VP*, mol m^−1^ s^−1^) were calculated by Equation (4) and Equation (5). The ratio of water to DMMP or CEPS vapor permeation was defined as selectivity and calculated by Equation (6). Three parallel tests of each membrane were conducted to ensure the accuracy of the results [14].

(4) VTR=W/(t×A)

(5) VP=VTR×L

(6) $$S=VP_{\mathrm{water}}/VP_{\mathrm{simulants}}$$

where *t* represents the time, *A* represents the samples area to be tested, and *L* *is* the thickness of sample.

## 3. Results

### 3.1. Synthesis and Characterization of PVDF-g-SSS

**Scheme 1:**
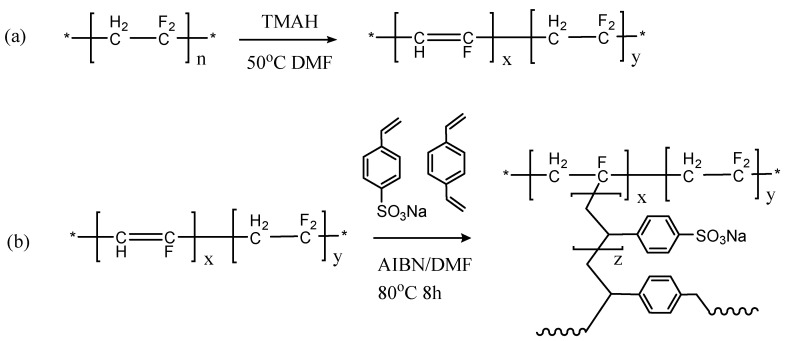
Reaction scheme for synthesizing of (**a**) Modified PVDF and (**b**) PVDF-*g*-SSS.

The PVDF-*g*-SSS membrane was prepared by a one-pot reaction. Firstly, TMAH methanol (10 wt%) solution was used to partially eliminate HF from PVDF and generate carbon–carbon double bonds (Scheme 1a). Then the SSS monomer was grafted to the modified PVDF via free radical polymerization initiated by AIBN (Scheme 1b). The volatile solvent was evaporated at 70 °C to obtain the PVDF-*g*-SSS membrane.

**Figure 3:**
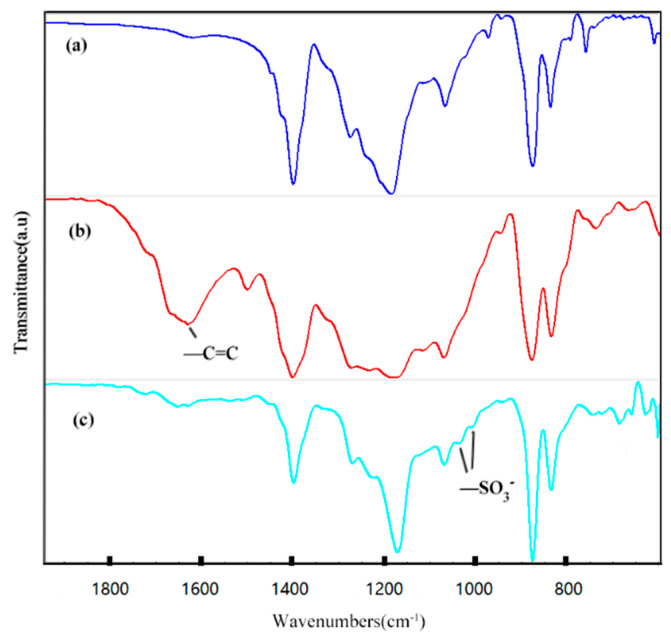
FT-IR spectra of (**a**) PVDF, (**b**) modified PVDF, and (**c**) PVDF-*g*-SSS.

Figure 3 depicted the FT-IR spectra of pure PVDF and TMAH treated PVDF. Compared with pure PVDF (a), the TMAH treated PVDF (b) shows a new band at 1610 cm^−1^ is representative of the stretching vibration of the carbon–carbon double bond, indicating the elimination of HF by TMAH and generation of carbon–carbon double bond active site. In the FTIR spectrum of the PVDF-*g*-SSS membrane, absorption bands at 1007 and 1034 cm^-1^ are representative of the symmetric telescopic vibration of sulfonate [36], suggesting the successful grafting of SSS onto the PVDF backbones, as shown in Scheme 1.

**Figure 4:**
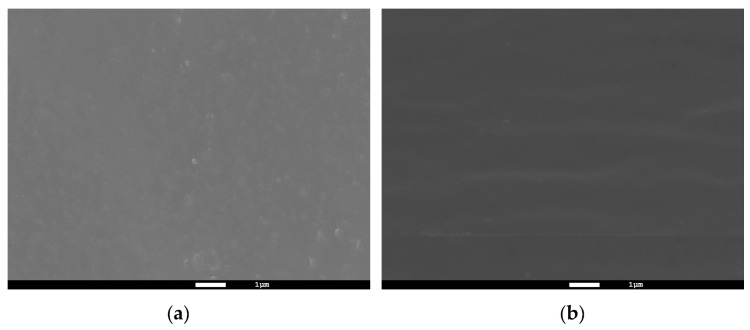
SEM image of (**a**) surface, (**b**) cross-section of M3.

The SEM image in Figure 4 shows the surface and cross-section morphology of M3. It can be seen from the figure that the membrane material is uniform, dense, and non-porous. The morphologies of other membrane samples are similar to M3, which is also uniformly dense.

### 3.2. IEC, WU, and LSR

IEC is a crucial property of a PEM, which decides the WU, LSR, water/DMMP, and water/CEPS selectivity. As shown in Table 2, the IEC values increase from M-1 to M-6, which can be explained by the increasing grafting content of SSS. It is also observed that the WU and LSR increase with increasing IEC in a nonlinear manner. From 0 to 1.73 mmol/g, the WU and LSR increase with IEC gradually. Afterward, for M4 to M6, the WU and LSR leaped from 23.1% to 77.4% and 8.3% to 28.3%, respectively.

### 3.3. Water Contact Angle

**Figure 5:**
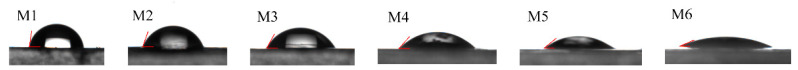
The water contact angle of M1–M6 membranes.

Figure 5 and Table 2 shows the water contact angle of synthesized membranes. The water contact angle decrease from M1 to M6 due to higher surface hydrophilicity at high IEC, which is coherent to the trend of WU. These results proved that the hydrophilicity could be improved by grafting modification.

### 3.4. Mechanical Properties

**Figure 6:**
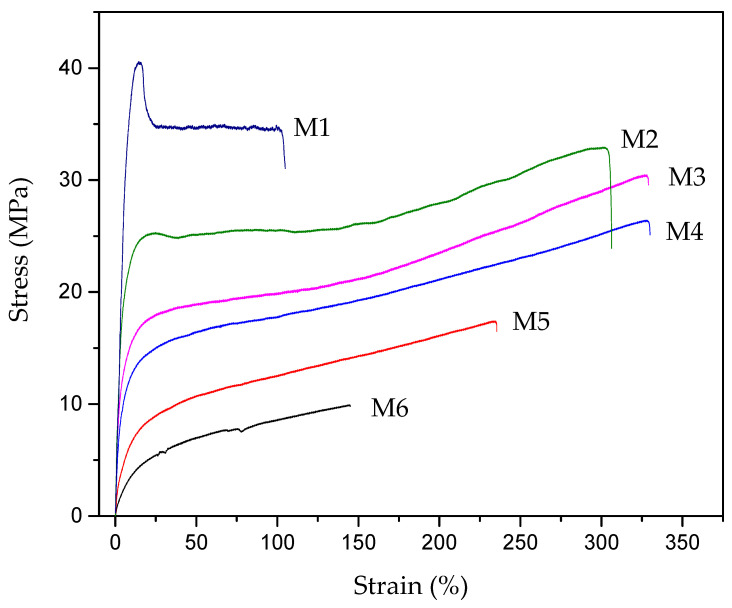
Typical stress–strain curves of M1–M6 membranes.

Excellent mechanical properties of the membrane are crucial for CPC application. Figure 6 shows the stress–strain curves of M1–M6 membranes. It is clearly observed that the tensile strength decrease with increasing IEC. The elongation-at-break first increases from M1 to M4 and then decreases. The tensile strength and elongation-at-break of prepared membranes are shown in Table 3. Compared with the unmodified PVDF membrane (M1), the membrane flexibility, i.e., elongation-at-break, is significantly improved by grafting modification. The decrease in tensile strength is due to the stronger plasticizing effect of water at higher WU. The as-synthesized membrane showed tensile strength sufficient for CPC application.

### 3.5. Permeations and Selectivities of Membranes

**Figure 7:**
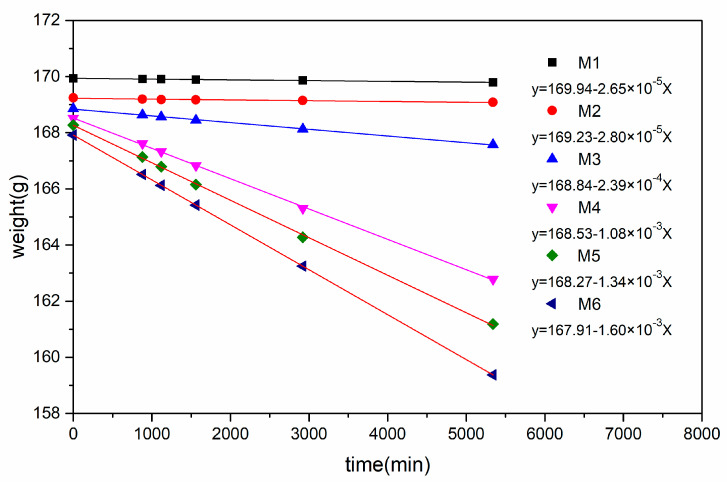
Typical weight–time curves for measuring VTR (water) of M1–M6.

To prevent severe heat stress and the injury caused by it, the water vapor transfer rate (WVTR) should be above 2000 g m^−2^ day^−1^ in chemical protection clothing applications [5]. The sodium sulfonate groups in PVDF-*g*-SSS have a good affinity to water molecules, thus can provide hydrophilic pathways to promote water diffusion and improve the breathability of CPC. In this study, the water permeability was measured by ASTM E96-95 method. As shown in Figure 7 and Table 4, the water vapor transfer rate increased from 60 to 3668 g m^−2^ day^−1^ as the IEC increased from 0 to 2.29 mmol/g. Besides, an obvious leap in WVTR was observed from M3 to M4, which may be due to the formation of a continuous water transport channel at a higher content of SSS groups. The WVTR is also depicted in Table 4; M4, M5, and M6 with WVTR above 2000 can fulfill the requirement for CPC application [5].

**Figure 8:**
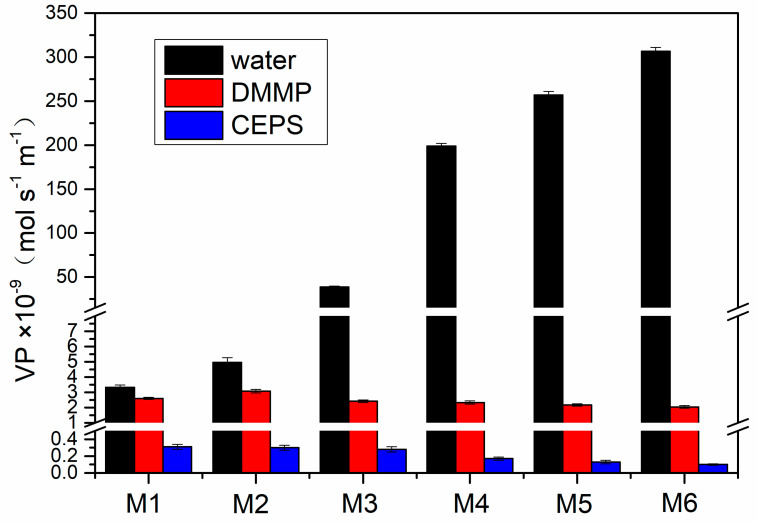
Vapor permeation for water, DMMP, and CEPS for M1–M6.

**Figure 9:**
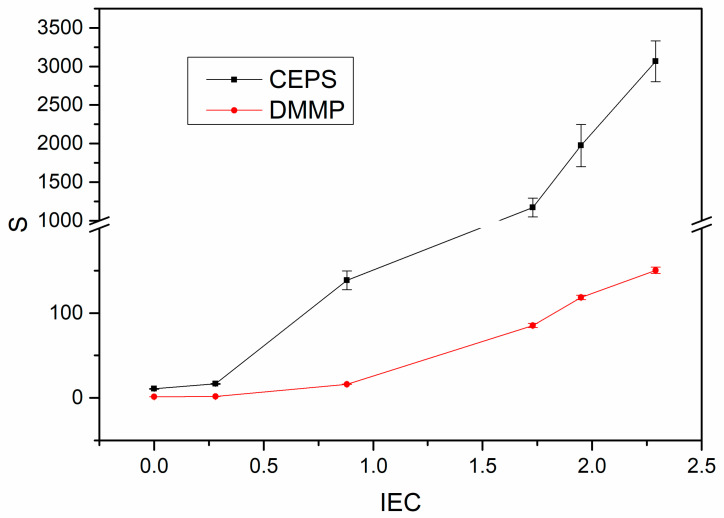
Selectivities of water over DMMP and CEPS for M1–M6.

The vapor permeation (VP) of dimethyl methyl phophonate (DMMP, a simulant for nerve agents) was measure using the same method as WVTR, and the results are shown in Figure 8 and Table 4. A decreasing trend of DMMP vapor permeation was observed as IEC increases from 0 to 2.29 mmol g^−1^. The selectivity between water vapor was calculated by S = VP_water_/VP_DMMP_. As shown in Figure 9, when the IEC is below 1 mmol/g, the selectivity increases gradually from 1.28 to 16.08. Significantly enhanced selectivity could be achieved when the IEC exceed 1.5 mmol g^−1^, e.g., 118.35 at 1.95 mmol g^−1^ and 150 at 2.29 mmol g^−1^, which are higher than that of a polystyrene sulfonic acid membrane (80) and a sulfonated poly(styrene-isobutylene-styrene) membrane (35) [14,19].

The vapor permeation of 2-chloroethyl phenyl sulfide (CEPS, a simulant for blister agents) was measured using the same method as DMMP, and the results are shown in Figure 8 and Table 4. A decreasing trend of CEPS vapor permeation similar to DMMP was observed with increasing IEC values. The selectivity between water vapor was calculated by S = VP_water_/VP_CEPS_. As shown in Figure 9, when the IEC is below 1 mmol g^−1^, the selectivity increases gradually from 10.71 to 138.43. Significantly enhanced selectivity could be achieved when the IEC exceeds 1.5 mmol g^−1^, e.g., 1975.69 at 1.95 mmol g^−1^ and 3066.70 at 2.29 mmol g^−1^, which are much higher than DMMP and also higher than that of a polystyrene sulfonic acid membrane (1500) and Nafion (400) [14].

## 4. Discussion

Nafion and polystyrene sulfonic acid (PSSA) are the state-of-the-art membrane materials for CPC. However, Nafion is too expensive for large scale applications, and PSSA does not possess sufficient mechanical properties. In this study, PVDF-*g*-SSS membranes were prepared using a convenient, low cost, and one-pot method. A series of PVDF-*g*-SSS membranes with different SSS grafting ratios were synthesized (M1 to M6). The successful grafting of SSS onto modified PVDF was clearly demonstrated by FT-IR. SEM images clearly show the morphologies of membranes were uniform, dense, and nonporous. This indicates that the selectivity of the membrane is not due to aperture screening but a dissolution–diffusion mechanism.

IEC measurement suggests a high conversion rate of SSS monomer (>70%). The WU and LSR were found to increase with IEC in a nonlinear manner. For M5 and M6 with IEC of 1.95 mmol/g and 2.29 mmol/g, an obvious leap in WU and LSR was observed. Mechanical properties tests suggest that the membrane flexibility is significantly improved by grafting modification, but tensile strength degrades obviously when the IEC exceeds 2.29 mmol/g (M6). The results show that the mechanical properties and dimensional stability of membrane materials with IEC lower than 2 mmol g^−1^ are controlled within an acceptable range, which is due to the cross-linking structure of membranes. Moderate cross-linking can limit the movement of the chain segments and the swelling of the membrane [37,38]. The contact angle measurement shows that hydrophilicity could be improved by increasing the content of the hydrophilic sodium sulfonate group. The increase of hydrophilic sodium sulfonate groups increases the surface energy of the material and enhances the water wettability of the membrane. Furthermore, water wettability is crucial for sweat permeation, thus directly influencing the comfort of CPC.

The WVTR test shows that the water vapor transfer rate of membranes increase with increasing IEC. From M3 to M4, the WVTR shows an obvious leap, which may be due to the formation of a continuous water transport channel at high SSS content. As well known, due to electrostatic interaction, the branch chains contained hydrophilic sulfonic acid group will gather together to form ion clusters. When being hydrated, ion clusters will swell and interconnect to form hydrophilic channels, thus accelerating the transfer of water. The increase of the sulfonic acid group content is beneficial to the increase of the number and size of ion clusters, thus accelerating the formation of water channels [39,40,41]. Membranes with IEC > 1.7 mmol g^−1^ shows WVTR above 2000 g m^−2^ day^−1^, which can fulfill the requirement of CPC application. DMMP vapor permeation (VP) test shows the VP of DMMP and CEPS decrease with IEC values, which is opposite to that of water vapor permeation. As a result, high water/DMMP selectivity >150 could be achieved at the IEC of 2.29 mmol/g, and high water/CEPS selectivity >3000 could be also achieved at 2.29 mmol/g.

This excellent selectivity at high IEC values may be explained by the formation of an interconnected SSS phase, which allows the transport of water and blocks the transport of DMMP. Vapor permeation occurred via two processes: (a) vapors sorption process and (b) vapors diffusion process [42]. The SSS groups in PVDF-*g*-SSS can form hydrated ionic regions, i.e., a water channel, which can lead to good absorption ability to water molecules. On the other hand, the hydrophobic CWA simulants would be repelled by the water channels, resulting in low absorption. In the diffusion process, water mainly diffuses through the hydrophilic phase, while DMMP and CEPS mainly diffuse through the hydrophobic phase [10]. With the increasing SSS content, the fraction of hydrophilic phase increases, and the fraction of hydrophobic phase decreases, thus resulting in higher water/DMMP and water/CEPS selectivities. Within an IEC range from 1 to 2 mmol/g, the apparent leap in selectivity could be due to the evolved interconnected hydrophilic phase and discontinuous hydrophobic phase. The much higher selectivity of water/CEPS than that of water/DMMP may be explained by worse hydrophilicity and lower vapor pressure at 35 °C of CEPS [5]. Moreover, the overall performance of the PVDF-*g*-SSS membrane has certain advantages compared with membranes of other studies. The membrane has superior selectivity, sufficient mechanical properties, and WVTR. The detailed comparison results are shown in Table 5.

## 5. Conclusions

The above results showed that the PVDF-*g*-SSS membrane holds the potential for CPC application, and the membrane IEC determines the overall performance. High IEC benefits WVTR and selectivity but causes decreased dimensional stability and mechanical properties in a high humidity environment. On the contrary, low IEC membranes have excellent dimensional stability and mechanical properties but lower WVTR and selectivity. Therefore, the IEC must be carefully optimized to achieve a balanced overall performance in CPC application. For the PVDF-*g*-SSS membrane developed in this study, the optimal IEC range is about 1.5–2 mmol/g, which can deliver a tensile strength of 17.4 MPa, WVTR of 3072, water/DMMP selectivity of 118, and water/CEPS selectivity of 1976.

## Figures and Tables

**Table 1:** Compositions of membranes with different IECs.

Sample	PVDF (g)	SSS (g)	DVB (g)	AIBN (g)	DMF (mL)
M1	20	0	0	0	200
M2	20	2.5	0.25	0.025	225
M3	20	6	0.6	0.06	260
M4	20	10.4	1.04	0.104	304
M5	20	16	1.6	0.16	360
M6	20	24	2.4	0.24	440

**Table 2:** Water content, ion-exchange capacity, linear swelling ratio, and water contact angle.

Sample	IEC (mmol/g)	WU (%)	LSR (%)	CA of Water (^o^)
M1	0	1.3	0	81.9 ± 3.5
M2	0.28	8.3	1. 7	70.9 ± 2.8
M3	0.88	14.3	5.0	61.2 ± 2.5
M4	1.73	23.1	8. 3	48.0 ± 2.6
M5	1.95	53.4	16. 7	39.3 ± 2.3
M6	2.29	77.4	28. 3	23.6 ± 1.9

**Table 3:** The thickness and mechanical properties of PVDF-*g*-SSS membranes.

Sample	Thickness (μm)	Strength (MPa)	Elongation-at-Break (%)
M1	85	40.6 ± 2.1	112.0 ± 12.8
M2	120	32.9 ± 2.3	306.3 ± 24.2
M3	110	30.5 ± 2.6	329.0 ± 29.2
M4	125	26.4 ± 1.9	329.9 ± 25.7
M5	130	17.4 ± 2.0	235.5 ± 18.3
M6	130	9.9 ± 1.1	145.1 ± 13.5

**Table 4:** VTR of water and DMMP, VP of water and DMMP, and selectivities of M1–M6 membranes.

Sample	VTR of Water (g m^−2^ day^−1^)	VP of Water ×10^−9^ (mol s^−1^ m^−1^)	VP of DMMP ×10^−9^ (mol s^−1^ m^−1^)	VP of CEPS × 10^−9^ (mol s^−1^ m^−1^)	Selectivity (Water/DMMP)	Selectivity (Water/CEPS)
M1	60 ± 3	3.32 ± 0.16	2.60 ± 0.09	0.31 ± 0.03	1.28 ± 0.03	10.71 ± 0.52
M2	64 ± 4	4.95 ± 0.31	3.07 ± 0.15	0.30 ± 0.03	1.61 ± 0.04	16.5 ± 0.62
M3	548 ± 15	38.76 ± 1.06	2.41 ± 0.10	0.28 ± 0.03	16.08 ± 0.16	138.43 ± 11.05
M4	2476 ± 34	199.04 ± 2.73	2.33 ± 0.12	0.17 ± 0.02	85.42 ± 2.49	1170.82 ± 121.69
M5	3072 ± 47	256.84 ± 3.93	2.17 ± 0.10	0.13 ± 0.02	118.35 ± 2.55	1975.69 ± 273.72
M6	3668 ± 54	306.67 ± 4.35	2.04 ± 0.10	0.10 ± 0.01	150.32 ± 3.76	3066.70 ± 263.17

**Table 5:** Comparison of recently reported CPC selectively permeable membranes

Membrane	IEC (mmol/g)	Strength (MPa)	VTR of Water (g m^−2^ Day^−1^)	Selectivity (Water/DMMP)	Selectivity (Water/CEPS)	Reference
PSS-nonwoven	-	9	4500	80	1500	[14]
Nafion-117	0.89	43	-	66	340	[14]
SIBS-g-PVPA	1.8	-	-	2.4	-	[16]
SIBS 61-CNT0.5	1.41	-	-	32	-	[17]
SIBS84-IP 1	2.2	-	-	50	-	[18]
SIBS-30-100k-84.4-Ba	-	-	-	35	-	[19]
